# EFICAz^2^: enzyme function inference by a combined approach enhanced by machine learning

**DOI:** 10.1186/1471-2105-10-107

**Published:** 2009-04-13

**Authors:** Adrian K Arakaki, Ying Huang, Jeffrey Skolnick

**Affiliations:** 1 Center for the Study of Systems Biology, School of Biology, Georgia Institute of Technology, Atlanta, Georgia, 30318, USA; 2 California Institute for Telecommunications and Information Technology, University of California, San Diego, La Jolla, CA, 92093, USA

## Abstract

**Background:**

We previously developed EFICAz, an enzyme function inference approach that combines predictions from non-completely overlapping component methods. Two of the four components in the original EFICAz are based on the detection of functionally discriminating residues (FDRs). FDRs distinguish between member of an enzyme family that are homofunctional (classified under the EC number of interest) or heterofunctional (annotated with another EC number or lacking enzymatic activity). Each of the two FDR-based components is associated to one of two specific kinds of enzyme families. EFICAz exhibits high precision performance, except when the maximal test to training sequence identity (MTTSI) is lower than 30%. To improve EFICAz's performance in this regime, we: i) increased the number of predictive components and ii) took advantage of consensual information from the different components to make the final EC number assignment.

**Results:**

We have developed two new EFICAz components, analogs to the two FDR-based components, where the discrimination between homo and heterofunctional members is based on the evaluation, via Support Vector Machine models, of all the aligned positions between the query sequence and the multiple sequence alignments associated to the enzyme families. Benchmark results indicate that: i) the new SVM-based components outperform their FDR-based counterparts, and ii) both SVM-based and FDR-based components generate unique predictions. We developed classification tree models to optimally combine the results from the six EFICAz components into a final EC number prediction. The new implementation of our approach, EFICAz^2^, exhibits a highly improved prediction precision at MTTSI < 30% compared to the original EFICAz, with only a slight decrease in prediction recall. A comparative analysis of enzyme function annotation of the human proteome by EFICAz^2 ^and KEGG shows that: i) when both sources make EC number assignments for the same protein sequence, the assignments tend to be consistent and ii) EFICAz^2 ^generates considerably more unique assignments than KEGG.

**Conclusion:**

Performance benchmarks and the comparison with KEGG demonstrate that EFICAz^2 ^is a powerful and precise tool for enzyme function annotation, with multiple applications in genome analysis and metabolic pathway reconstruction. The EFICAz^2 ^web service is available at:

## Background

From a purely biochemical point of view, enzymes constitute the most important group of proteins. They are versatile, catalyzing most chemical reactions involved in the metabolism of living organisms, and abundant, representing approximately 15% to 35% of a given proteome [1,2]. Enzymes are classified according to the Enzyme Commission (EC) system, a hierarchical system that assigns a unique four-field number to each enzymatic activity [3]. The first field of an EC number indicates the general class of catalyzed reaction. The second and third fields depend on different criteria related to the chemical features of the substrate and the product of the reaction, and the fourth field is a sequential number without any special meaning. A comprehensive and detailed enzyme function annotation of the available genomes is necessary not only to increase our understanding of the biochemistry of living organisms, but also to gain more insight into the evolutionary processes that originated the diversity of enzymes currently found in nature [4]. The precise assignment of EC numbers to catalytic proteins is a vital requirement for the correct reconstruction of metabolic pathways [5]. Moreover, reconstructed metabolic pathways play a key role in many biomedical approaches [6-9], but the success of these applications strongly depends of the quality of the functional annotations of the enzymes comprising such pathways [10].

Despite the great importance of precise EC number assignments, enzyme functions as well as other molecular, cellular or physiological functions, are often inferred from sequence similarity to previously characterized proteins [11]. In this annotation modality, commonly known as "prediction by homology transfer", the (incorrect) assumption is that all homologs have the same function [12]. This functional annotation strategy is negatively affected by at least two factors. The first factor is the functional diversity of highly similar sequences observed in many protein families [13]. For example, to transfer detailed enzyme function, given by four-field EC numbers, with an average precision of at least 90%, a sequence identity threshold of 60% is required [14]. However, the functional annotation of many genomes has been carried out employing much lower thresholds [15]. The second factor is the structural and functional modularity of proteins [16]; thus, when the modular nature of proteins is disregarded, functional annotations based on best database hits are often erroneous [17]. Mainly due to these factors, sequence similarity-based annotation strategies result in a high number of errors [18,19] that often propagate in public databases [20]. For instance, it has been estimated that functional assignments inferred by sequence similarity in the Gene Ontology sequence database (GOSeqLite), have an estimated error rate of 49% [21]. Other approaches for enzyme function prediction do not directly depend on the level of similarity between sequences. For example, several methods are based on the identification of specific structural patterns associated with functional sites [22-24], but they are limited by the requirement that the query protein's structure be solved. Yet other approaches are based on the analysis of properties of proteins such as tissue specificity, subcellular location and phylogenetic information [25], or genome context and other functional association evidence [26]. However, these methods also suffer from the lack of consistent and comprehensive database annotations related to this kind of sequence-independent features.

To address the limitations of transfer of enzyme function by sequence similarity, we developed EFICAz (Enzyme Function Inference by a Combined Approach), an engine for large-scale high-precision enzyme function inference [27]. The original implementation of EFICAz combines the predictions of four independent methods: (C1) **CHIEFc family based FDR recognition**: detection of Functionally Discriminating Residues (FDRs) in enzyme families obtained by a Conservation-controlled HMM Iterative procedure for Enzyme Family classification (CHIEFc), (C2) **Multiple Pfam family based FDR recognition**: detection of FDRs in combinations of Pfam families that concurrently detect a particular enzyme function, (C3) **CHIEFc family specific SIT evaluation**: pairwise sequence comparison using a CHIEFc family specific Sequence Identity Threshold (SIT), and (C4) **High specificity multiple PROSITE pattern recognition**: detection of multiple PROSITE patterns that, taken all together, are specifically associated to a particular enzyme function. Since each predictive component was designed to be highly precise and predictions made by any pair of components do not completely overlap (including C1 and C2, which only differ in the way the protein families are defined), at the final stage, EFICAz makes a particular EC number assignment when one or more of the four component methods predict a given EC number. Since EFICAz and its components have been fully described before [27], here, we briefly introduce the basics of the predictive components based on the recognition of FDRs and highlight possible improvements.

A CHIEFc or Pfam enzyme family *E *is defined by a multiple alignment of sequences evolutionary related to a seed group of sequences sharing a particular EC number EC_*E*_. FDRs are residues in specific positions of the alignment, selected via an Evolutionary Footprinting method [27] for their ability to discriminate between homo-functional and hetero-functional family members. Homo- and hetero-functional family members are defined as sequences annotated or not annotated with the EC number EC_*E*_, respectively. To apply an FDR recognition method, we first determine if a query sequence **q **is a member of an enzyme family *E *by evaluating a Hidden Markov Model derived from *E*. If so, we check if **q **exhibits conservation of the FDRs associated with *E*. When both conditions are fulfilled, we predict that **q **is a homo-functional member of *E *and assign the EC number EC_*E *_to the query sequence **q**. A figure illustrating the concept of FDRs can be found in Additional file 1: Figure S1. Example of Functionally Discriminating Residues (FDRs). A potential pitfall of the FDR recognition methods is that if the number of FDRs for a given enzyme family is too small, it can be difficult to achieve high prediction precision, because the matching of a very small number of residues in an alignment is more likely to occur by chance. Conversely, if the number of FDRs is too large, the prediction recall might suffer, because the matching of a large number of residues in an alignment imposes a very restrictive condition. In principle, these issues could be addressed by techniques more advanced than FDR matching in terms of their ability to detect the signals characteristic of homo-functional enzyme family members in the query sequence. In this work, we describe the development of a method for enzyme function inference that is based on this premise. We employ a Support Vector Machine (SVM) learning approach [28] that evaluates all the aligned positions between a query sequence and the multiple sequence alignment associated to a given Pfam or CHIEFc enzyme family. We term these components: (C5) **CHIEFc family based SVM evaluation **and (C6) **Multiple Pfam family based SVM evaluation**, and our benchmarks show that they yield higher predictive performance than their counterparts based on FDR recognition.

As mentioned above, in the previous implementation of EFICAz, all EC numbers predicted by the four original component methods were been reported, whether they agreed with each other or not. Here, based on estimations of the method's performance that are more realistic than those published before [1,27], we show that such a strategy tends to negatively affect prediction precision, especially at low levels of maximal test to training sequence identity (MTTSI, formally defined in the Methods section). To address this issue, we have developed a tree-based classification algorithm [29] that applies a set of hierarchical rules to generate an EC number assignment from the list of the component methods that predict such EC number and the query sequence's MTTSI. We have included the two additional SVM-based component methods as well as the classification tree algorithm in the current implementation of EFICAz, that we term EFICAz^2^. According to the results of our performance benchmarks, EFICAz^2 ^is dramatically more precise than EFICAz at low MTTSI, while it shows only a modest decrease in recall in this MTTSI regime.

The rest of this paper is organized as follows: in the Results and Discussion section, we describe the development and benchmarking of the SVM-based enzyme function inference method and the classification tree algorithm to generate the final EC number prediction, and present a comparative study of enzyme function annotations of the human proteome by EFICAz^2 ^and KEGG [30]. In the Conclusions section, we summarize the present work, stress its significance, and discuss its limitations. Finally, in the Methods section, we describe the data sources and procedures for training and benchmarking of EFICAz^2^, provide details about the statistical analyses and technical aspects of the generation of SVM and classification tree models, and describe the data sources for the comparative analysis of enzyme function annotation of the human proteome.

## Results and Discussion

### Novel EFICAz components based on SVM

Two of the four component methods in the original implementation of EFICAz are based on the identification of homo-functional members of a given CHIEFc (C1) or Multiple Pfam enzyme family (C2), i.e., members whose enzymatic activity coincides with that of the seed enzymes that originated the family. The criterion followed by these methods to consider a query sequence as homo-functional (and therefore make the corresponding EC assignment) is the matching of FDRs. Since FDRs constitute a subset of all residues in the multiple sequence alignment associated to an enzyme family, we reasoned that an algorithm operating over all the aligned positions (i.e., with access to all possible information) could achieve higher discriminatory power, at least in certain cases. This situation is analogous to that of patterns and profiles for the identification of protein families and domains in the PROSITE database [31].

Initially, PROSITE consisted of patterns alone and was later enriched by the inclusion of profiles. Although, in general, PROSITE profiles exhibit increased sensitivity with respect to patterns, profiles and patterns complement each other, i.e. both types of descriptors offer unique advantages in particular cases [32].

Our implementation of the profile-like approach to the recognition of homo-functional sequences is based on SVM models associated to each enzyme family. The basic idea of the SVM algorithm is mapping the data from an input space into a high-dimensional feature space via a kernel function, and finding a hyper-plane to separate positive and negative samples in the feature space [28]. The training of the SVM models is carried out using the whole set of aligned residues in the corresponding multiple sequence alignment, which include both positives or homo-functional sequences and negatives or hetero-functional sequences (see Methods section, "Support vector machine models"). The new component methods were termed: (C5) CHIEFc family based SVM evaluation and (C6) Multiple Pfam family based SVM evaluation. In order to compare the performance of the new SVM-based components to that of the FDR-based components, we carried out extensive benchmarking. First, we trained the two FDR-based (C1 and C2) and the two SVM-based components (C5 and C6) using previous releases of the corresponding databases; these specific versions of the component methods were later included in EFICAz^2 ^version 10, based on the Release 10 of UniProt [33] (see Methods section, "Datasets for the training of different EFICAz^2 ^versions"). Then, we selected test sequences from all of the well annotated, newly added Swiss-Prot sequences in UniProt Release 12.6 that were not included in the Release 10. Finally, for each test sequence, we collected the enzyme function predicted by each of the four components under evaluation and calculated the average precision and recall (see Methods section, "Benchmarking of EFICAz^2 ^version 10"). The statistical significance of the differences in method's performance was evaluated as described in "Statistical analyses", in the Methods section.

**Figure 1:**
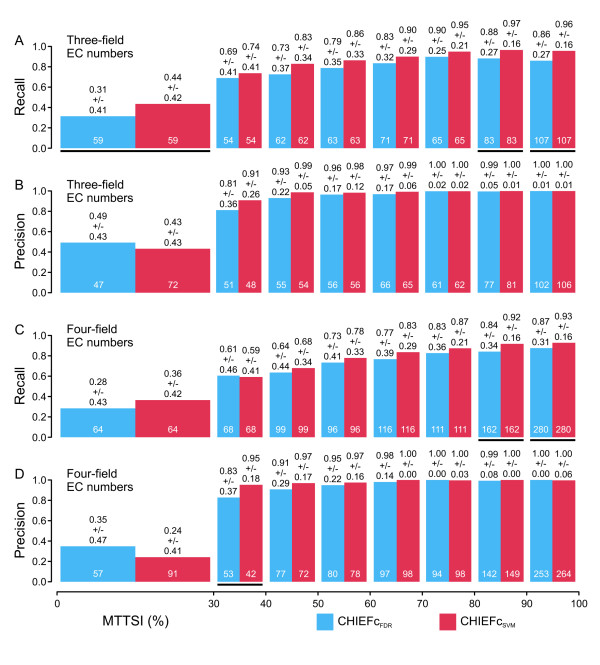
**Prediction performance of the FDR-based and SVM-based approaches applied to Multiple Pfam enzyme families**. For three-field (A, B) or four-field EC number classifiers (C, D), the average recall (A, C) and average precision (B, D) of the FDR-based (blue columns) and SVM-based (red columns) approaches is plotted at different intervals of maximal test to training sequence identity (MTTSI). The average of each performance indicator is done over all the EC numbers defined in the specified MTTSI interval (numbers at the bottom of each column). Details about the benchmark can be found in "Benchmarking of EFICAz^2 ^version 10", in the Methods section. Statistically significant differences in performance are indicated by black lines under the corresponding columns (see "Statistical analyses", in the Methods section). Values on top of each column represent average +/- standard deviation.

**Figure 2:**
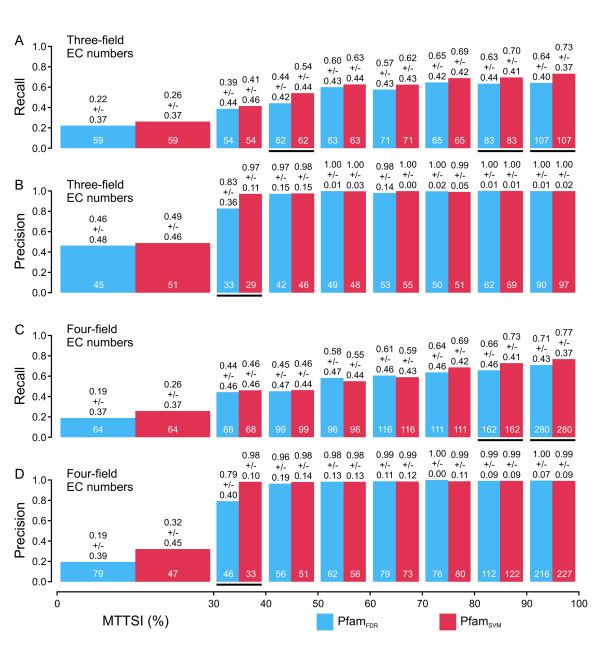
**Prediction performance of the FDR-based and SVM-based approaches applied to CHIEFc enzyme families**. For three-field (A, B) or four-field EC number classifiers (C, D), the average recall (A, C) and average precision (B, D) of the FDR-based (blue columns) and SVM-based (red columns) approaches is plotted at different intervals of maximal test to training sequence identity (MTTSI). The average of each performance indicator is done over all the EC numbers defined in the specified MTTSI interval (numbers at the bottom of each column). Details about the benchmark can be found in "Benchmarking of EFICAz^2 ^version 10", in the Methods section. Statistically significant differences in performance are indicated by black lines under the corresponding columns (see "Statistical analyses", in the Methods section). Values on top of each column represent average +/- standard deviation.

Figure 1 shows a comparison of the performance of the FDR-based (C1) and the SVM-based approaches (C5) applied to three-field EC number (Figure 1AB) and four-field EC number CHIEFc enzyme families (Figure 1CD). In the case of three-field EC number classifiers, the SVM-based method achieves significantly higher average recall at MTTSI lower than 30% and higher than 80% (Figure 1A), but shows no significant difference in average precision (Figure 1B). The SVM-based implementation for four-field EC number classifiers also shows an advantage in terms of average recall at MTTSI higher than 80% (Figure 1C), in addition to a significant increase of average precision at MTTSI between 30% and 40%. Figure 2 shows a comparison of the performances of the FDR-based (C2) and the SVM-based approaches (C6) applied to three-field EC number (Figure 2AB) and four-field EC number Multiple Pfam enzyme families (Figure 2CD). For three-field EC number classifiers, the SVM-based method exhibits significantly higher average recall in the 40% to 50% and higher than 80% MTTSI intervals (Figure 2A), and significantly higher average precision in the 30% to 40% MTTSI interval (Figure 2B). For four-field EC number classifiers, the improvements in average recall (Figure 2C) and precision (Figure 2D) of the SVM-based approach applied to Multiple Pfam families occur in the same MTTSI intervals as the improvements observed when this approach is applied to CHIEFc families (Figure 1C, D). In summary, in all the cases where the differences are statistically significant, the SVM-based methods show improved performance with respect to the corresponding FDR-based implementations. In fact, with only a few exceptions, the SVM-based methods exhibit the same or better average recall and precision than the FDR-based ones, although in several MTTSI intervals the current benchmark does not contain enough test sequences to make the differences between methods statistically significant.

**Figure 3:**
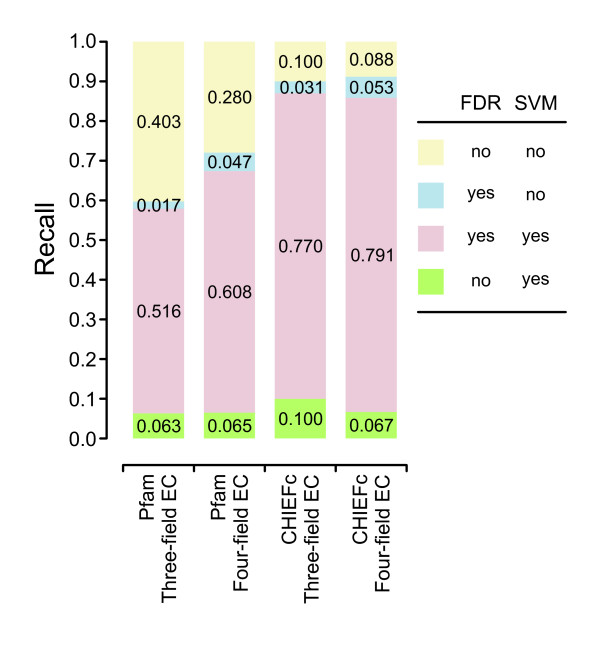
**Prediction overlap of FDR-based and SVM-based methods**. The fractions of test sequences (corresponding to the benchmark described in "Benchmarking of EFICAz^2 ^version 10", in the Methods section) correctly predicted by three or four-field EC number classifiers applied to Multiple Pfam or CHIEFc enzyme families are represented. For combination of enzyme family and level of description of the classifiers, we show the fraction corresponding to unique predictions made by the FDR-based (blue) or SVM-based method (green), and the fraction corresponding to predictions made by both (orange) or none of the methods (yellow).

Since EFICAz works by combining the predictions of different non-completely overlapping methods, even if the FDR- and the SVM-based approaches had identical average performance, they could still be both useful, provided that each method can generate its own set of unique predictions. Figure 3 shows the fraction of test sequences correctly predicted by either approach, both approaches, or none of them, when implemented on three-field or four-field EC number classifiers based on Pfam or CHIEFc enzyme families. Although the overlap of the approaches is high, each method provides a set of unique predictions, with a higher contribution from the SVM-approach for three-field EC number classifiers (10.0% and 6.3% for Multiple Pfam and CHIEFc enzyme families, respectively), and similar contributions from each approach for four-field EC number classifiers. Thus, we decided to keep the FDR-based predicted components and incorporate the SVM-based components: (C5) CHIEFc family based SVM evaluation and (C6) Multiple Pfam family based SVM evaluation in the new version of EFICAz.

### Combination rules based on classification trees

**Figure 4:**
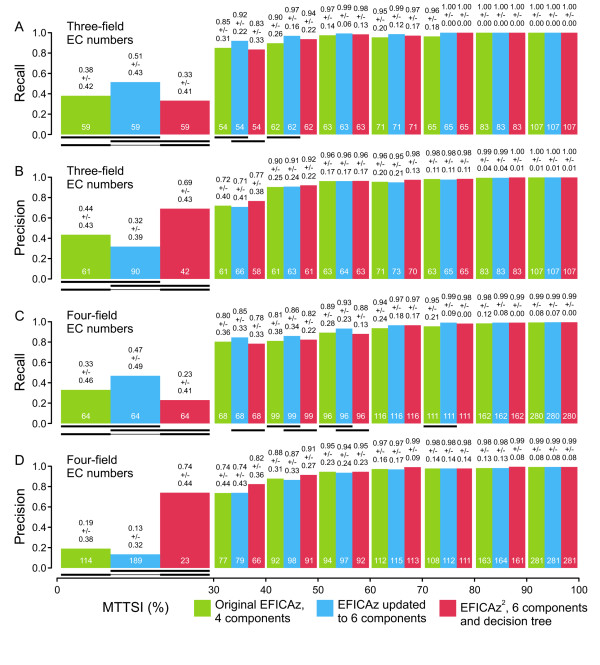
**Prediction performance of different EFICAz implementations**. For three-field (A, B) or four-field EC number classifiers (C, D), the average recall (A, C) and average precision (B, D) of the original EFICAz (green columns), EFICAz plus the new SVM-based components (blue columns) and EFICAz^2 ^(red columns) is plotted at different intervals of maximal test to training sequence identity (MTTSI). The average of each performance indicator is done over all the EC numbers defined in the specified MTTSI interval (numbers at the bottom of each column). Details about the benchmark can be found in "Benchmarking of EFICAz^2 ^version 10", in the Methods section. Statistically significant differences in performance are indicated by black lines under the corresponding columns (see "Statistical analyses", in the Methods section). Values on top of each column represent average +/- standard deviation.

The original version of EFICAz adopted the simple strategy of predicting a given EC number when at least one of its four component did [27]. Figure 4 shows the result of a benchmark that compares the performance of three different implementations of EFICAz (version 10), in terms of average recall (Figure 4AC) and average precision (Figure 4BD), distinguishing between two levels of detail of enzyme function given by three-field (Figure 4AB) or four-field EC numbers (Figure 4CD). As opposed to the results from previous benchmarks [1,27], the original EFICAz implementation shows poor average precision at MTTSI < 30% (Figure 4BD, green columns). The discrepancy arises because in this work we employed a more rigorous way to estimate the precision of our method (see Methods section, "Benchmarking of EFICAz^2 ^version 10"). We analyzed the effect of adding the two SVM-based components to EFICAz, bringing the total number of component methods to six (Figure 4, blue columns). As expected, a general pattern of increased recall (Figure 4AC) and decreased precision (Figure 4BD) with respect to the original four-component EFICAz can be observed, although only for three-field EC number classifiers at MTTSI < 30% was the decrease in precision statistically significant.

In order to improve the precision of our approach, we decided to investigate more efficient ways to integrate the predictions generated by the six EFICAz component methods. We had demonstrated in our previous work that increased precision can be achieved by requiring the consensus of two or more components of EFICAz [27]. Here, we decided to train decision tree models to find the optimal way to take advantage of consensual information from the different components. Decision trees are very effective tools in machine learning that produce accurate, highly interpretable predictions and have been successfully used in several computational biology and bioinformatics applications [34], including enzyme function prediction [25]. For our particular case, we sought decision trees able to output a binary outcome (whether a given EC number is assigned or not to a protein sequence), based on the prediction results of each component. Decision trees that produce discrete outcomes are called classification trees [29]. There are several possibilities to consider regarding the level of generalization of the classification trees, for example, whether or not they depend on the specific EC number type. In principle, EC number-specific classification trees could yield more accurate predictions. However, since not all the EC number types are represented in the set of test sequences, we opted for an EC number-independent solution.

**Figure 5:**
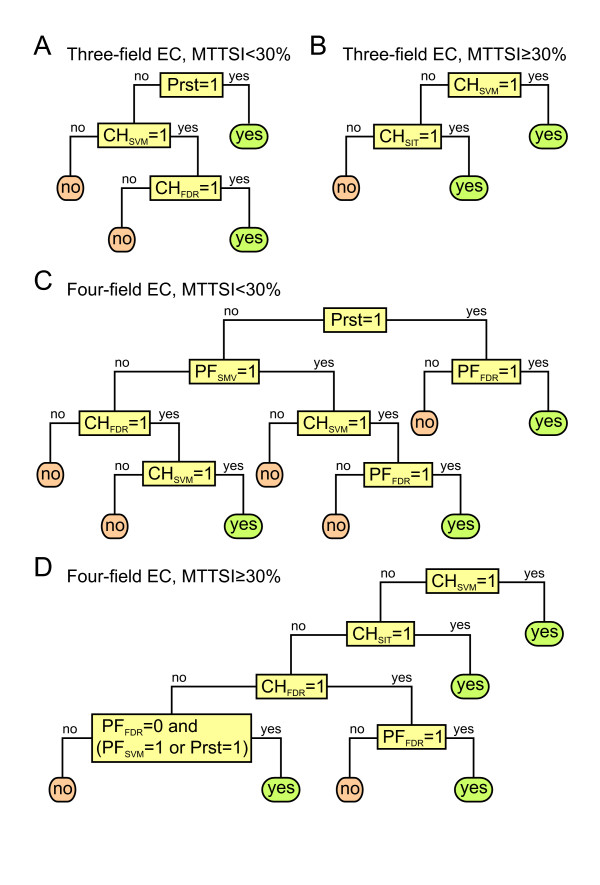
**Predictive models for EFICAz^2 ^based on classification trees**. Classification trees corresponding to three-field (A, B) and four-field EC numbers (C, D) to integrate predictions from each of the six EFICAz^2 ^components for protein sequences that exhibit MTTSI < 30% (A, C) or MTTSI ≥ 30% (B, D). CH_FDR _= CHIEFc family based FDR recognition; PF_FDR _= Multiple Pfam family based FDR recognition; CH_SIT _= CHIEFc family specific SIT evaluation; Prst = High specificity multiple PROSITE pattern recognition; CH_svm _= CHIEFc family based SVM evaluation; PF_svm _= Multiple Pfam family based SVM evaluation.

After the training procedure detailed in "Decision tree learning model" in the Methods section, we obtained the four classification trees shown in Figure 5, one for each combination of three or four-field EC number classifiers and low (< 30%) or high (≥ 30%) MTTSI. Inspection of the questions associated to the nodes of the classification trees indicates that the SVM-based components are the most informative ones, for example, CHIEFc family based SVM evaluation plays a role in all four trees (Figure 5). The version of our approach that employs these classification trees to integrate the information from the six possible component methods was termed EFICAz^2^.

We compared the performance of EFICAz^2 ^(Figure 4, red columns) to that of the original EFICAz with four components or the updated version with six components. Compared to the original EFICAz, EFICAz^2 ^displays a statistically significant decrease in average recall at MTTSI < 30% (a difference in recall of 5% and 10% for three- and four- field EC numbers, respectively, Figure 4AC) and at a few other MTTSI intervals, although the difference in recall is less than 5% in these latter cases. More importantly, EFICAz^2 ^shows a dramatic increase in average precision at MTTSI < 30% (a difference in precision of 25% and 55% for three- and four- field EC numbers, respectively, Figure 4BD). Similar tendencies, with average recall increases and average precision decreases of higher magnitude, can be observed when EFICAz^2 ^is compared to EFICAz updated to six components. In summary, we first shifted the precision-recall trade-off towards higher recall and lower precision by adding the SVM-based components to the original EFICAz implementation. Then, by making more efficient use of consensus between predictions from different components via classification tree models, we achieved acceptable levels of average precision at low MTTSI, with low impact on the average recall. The EFICAz^2 ^code is available upon request to academic and non-profit users. In addition, we have made EFICAz^2 ^available as a web service [35] that allows the submission of query protein sequences and returns the output via email. If an enzyme function inference is made, the output consists of the four-field or three-field EC number prediction/s, the predictive component/s that recognized the EC number/s, the MTTSI interval associated to the query sequence and the mean and standard deviation of the precision performance obtained from benchmarks.

EFICAz^2 ^exhibits an average precision of at least 90% for MTTSI ≥ 40% (Figure 4B, D), a non trivial achievement, considering that to achieve this level of precision from a sequence similarity criterion alone, MTTSI ≥ 60% is required [14]. Moreover, we significantly improved the prediction precision at MTTSI < 30%, compared to the original implementation of EFICAz. Nevertheless, the recall in this regime still requires additional improvement (average recall of 33% and 23% for three-field and four-field EC numbers at MTTSI < 30%, respectively, Figure 4AC). One possibility to overcome this EFICAz^2^'s limitation is to include methods that do not depend on sequence information. Some protein features that have been used before with the purpose of enzyme function prediction include protein- protein interaction [36], phylogenetic distribution, tissue specificity and subcellular localization [25]. Although we will explore the possibility of including non-sequence-dependent features of proteins in future versions of EFICAz, its implementation may be impaired by the low availability or inconsistency that this kind of annotations exhibits in current databases.

### Enzyme function annotation of the human proteome by EFICAz^2^

We carried out an enzyme function reannotation of the human proteome (24,305 protein sequences) using EFICAz^2 ^version 13 (see Methods section, "Datasets for the training of different EFICAz^2 ^versions") and compared our annotations with those available in a recent release of KEGG (see Methods section, "Enzyme function annotation of the human proteome"). We decided to use KEGG annotations rather than other sources to compare against our EFICAz^2 ^predictions because of the emphasis that this database puts on detailed EC number information, a fundamental requirement for the correct mapping of metabolic pathways. Two different levels of detail of the enzyme function assignment (given by three-field and four-field EC numbers) were considered separately for the analysis. Table 1 summarizes the results of the comparison. A single protein may have more than one enzymatic activity; therefore, multiple EC numbers can be assigned to the same protein. Where it is pertinent, both the number of protein sequences and the number of annotations (that can be higher than the number of sequences) were reported.

**Table 1:** Comparative enzyme function annotation of the human proteome^(1)^

Level of detail of the enzyme function assignment: Three-field EC numbers						
		EFICAz^2 ^predictions^(2)^				
Annotation source		EC numbers with less than three fields^(4)^: **20,889**	Three-field EC numbers: 3,508/**3,416**^(5)^			

	EC numbers with less than three fields^(4)^: **21,398**	**20,608**	EFICAz^2 ^novels: 798/**790**			
				Level of EC annotation agreement^(6)^		
				
KEGG annotations^(3)^			Annotation source	None	Partial	Full
			
	Three-field EC numbers: 2,954/**2,907**	KEGG novels: 309/**281**	EFICAz^2^	18/**18**	138/**67**	2,554/**2,541**
			KEGG	18/**18**	73/**67**	

Level of detail of the enzyme function assignment: Four-field EC numbers						

		EFICAz^2 ^predictions^(2)^				
		
Annotation source		EC numbers with less than four fields^(4)^: **21,660**	Four-field EC numbers: 2,850/**2,645**			

	EC numbers with less than four fields^(4)^: **21,833**	**21,350**	EFICAz^2 ^novels: 522/**483**			
				Level of EC annotation agreement^(6)^		
				
KEGG annotations^(3)^			Annotation source	None	Partial	Full
			
	Four-field EC numbers: 2,523/**2,472**	KEGG novels: 338/**310**	EFICAz^2^	49/**46**	260/**117**	2,019/**1,999**
			KEGG	46/**46**	120/**117**	

^(1) ^The source of the 24,305 human protein sequences is the KEGG Genes database Release 47.0+/06-26, of June 26, 2008.

^(2) ^Predictions made by EFICAz^2 ^version 13.

^(3) ^Annotations obtained from the KEGG Brite database Release 47.0+/06-26, of June 26, 2008.

^(4) ^Includes non-enzymes, considered as having zero-field EC numbers.

^(5) ^Non-bolded font indicates number of annotations while bolded font refers to the number of annotated protein sequences (a single protein can display more than one enzymatic activity, thus, multiple EC numbers can be assigned to the same protein sequence).

^(6) ^Here, we compare the agreement between annotations from KEGG and EFICAz^2 ^that have the same level of detail, whether three-field or four-field EC numbers. Three different levels of agreement are considered: 1) Full: all EC numbers assigned to the protein by KEGG and EFICAz^2 ^are identical, 2) Partial: at least one but not all the EC numbers assigned to the protein by KEGG and EFICAz^2 ^agree, and 3) None: none of the EC numbers assigned to the protein by KEGG and EFICAz^2 ^coincides.

Table 1 show that, although both KEGG and EFICAz^2 ^provide unique annotations, the novel assignments made by EFICAz^2 ^significantly exceed those from KEGG. At the level of detail of three-field EC numbers, there are 798 novel annotations by EFICAz^2 ^corresponding to 790 proteins versus 309 unique annotations for 281 proteins from KEGG. Similarly, for four-field EC numbers, there are 522 novel annotations for 483 proteins by EFICAz^2 ^versus 338 unique annotations for 310 proteins from KEGG. We analyzed the agreement between EFICAz^2 ^and KEGG assignments for the 2,626 sequences that were annotated with a level of detail of at least one three-field EC number by both sources. For a given annotated protein, we distinguished among three possibilities: i) full agreement, where all the EC number/s assigned to the protein by EFICAz^2 ^and KEGG coincide, ii) partial agreement, where at least one but not all the EC numbers assigned to the protein by these sources agree, and iii) no agreement, where none of the EC numbers assigned to the protein by these sources agree. For the 2,626 common sequences annotated with three-field EC numbers, the level of full agreement is 96.8%, while the level of partial agreement or better is 99.3%. Similarly, for the 2,162 sequences annotated with four-field EC numbers by both sources, the full and at least partial agreement is 92.5% and 97.9%, respectively. The matching of EC numbers is done at the stated level of detail, i.e. when comparing three-field or four-field EC numbers, only the first three fields or the full four fields are considered, respectively.

The level of agreement between KEGG and EFICAz^2 ^can also be assessed on the basis of the total number of EC number predictions by one or the other source, rather than by the total number of annotated proteins. The number of annotations and the number of proteins may differ because a single protein may have more than one enzymatic activity; therefore, more than one EC number may be associated to it. In this case, we only distinguish between agreement and lack of it. The number annotations by EFICAz^2 ^and KEGG for the 2,626 sequences annotated with three-field EC numbers by both sources is 2,710 and 2,645, respectively. Thus, the level of agreement is 96.7% ([67+2,554]/2,710) and 99.1% ([67+2,554]/2,645) when expressed in terms of the number of EFICAz^2 ^and KEGG three-field EC number annotations, respectively. The number of annotations by EFICAz^2 ^and KEGG for the 2,162 sequences annotated with four-field EC numbers by both sources is 2,328 and 2,185, respectively. Therefore, the level of agreement is 91.7% ([117+2,019]/2,328) and 97.8% ([117+2,019]/2,185), when expressed in terms of the number of EFICAz^2 ^and KEGG four-field EC number annotations, respectively.

This comparative analysis indicates that when both sources make EC number assignments for the same protein sequence, there is a high chance that these assignments are consistent. On the other hand, at the level of detail of three-field EC numbers, EFICAz^2 ^generates more than double the number of unique assignments (i.e., assignments for proteins annotated as non-enzymes by the other compared source), while it provides more than 50% additional unique assignments when four-field EC numbers are considered. The unique EC number assignments made by EFICAz^2 ^can be found in Additional file 2: Novel enzyme function annotations of the human proteome by EFICAz^2^.

## Conclusion

In this work, we described, implemented and tested EFICAz^2^, a new version of EFICAz [27], our automated approach for enzyme function prediction, enhanced by means of machine learning techniques. We increased the number of EFICAz components from four to six by adding two methods based on the evaluation of Pfam and CHIEFc enzyme families by SVM classifiers. The SVM-based components showed statistically significant performance improvements compared to their counterpart methods based on the detection of FDRs. We generated a set of classification trees to integrate and take advantage of the complementarity between the predictions from the six component methods, and achieved a remarkable increase in average precision at low MTTSI, with only moderate impact on average recall. When we applied EFICAz^2 ^to the enzyme function reannotation of the human proteome, we found that for proteins annotated as enzymes by both EFICAz^2 ^and KEGG, the assigned EC numbers were highly consistent. Moreover, the number of unique enzyme assignments generated by EFICAz^2 ^is significantly higher than the unique enzyme annotations in KEGG. Thus, the results of the performance benchmark and the comparison with KEGG, demonstrate that EFICAz^2 ^is a powerful and precise tool for enzyme function annotation, with multiple applications in genome analysis and metabolic pathway reconstruction.

## Methods

### Datasets for the training of different EFICAz^2 ^versions

The training of EFICAz^2 ^requires a source of protein sequences with high quality functional annotations; for this purpose, we employ the UniProt Knowledgebase database (UniProt) [33]. From the UniProtKB/Swiss-Prot component of UniProt (Swiss-Prot), we extract a set of enzyme sequences and a set of non-enzyme sequences, according to the criteria described in the original EFICAz article [27]. These reference sets are employed for the training of all the EFICAz^2 ^predictive components. Table 2 shows the number of sequences included in the "enzymes" and "non-enzymes" sets corresponding to versions 10 and 13 of EFICAz^2^, as well as the number of sequences with three- and four-field EC number annotations in the "enzymes" sets. To train EFICAz^2 ^versions 10 and 13, we used Releases 10 (March 2007) and 13 (February 2008) of UniProt, respectively. For training of the predictive components "Multiple Pfam family based FDR recognition" and "Multiple Pfam family based SVM evaluation" of both EFICAz^2 ^versions, we used the Pfam database [37] Release 22. Finally, for the training of the "High specificity multiple PROSITE pattern recognition" component of EFICAz^2 ^versions 10 and 13, we used the Releases 20.26 and 20.30 of the PROSITE database [31], respectively. For EFICAz^2 ^versions 10 and 13, Table 3 shows the number of Pfam enzyme families, CHIEFc enzyme families and PROSITE patterns as well as the number of different three-field and four-field EC numbers associated to them.

**Table 2:** Number of sequences in reference sets used for EFICAz^2 ^training

Reference sequence set	EFICAz^2 ^version 10	EFICAz^2 ^version 13
		
"non enzymes"	132.342	174,898
"enzymes" (all)	94,028	136,167
"enzymes" (three-field EC number)	90,801	131,503
"enzymes" (four-field EC number)	76,698	111,577

**Table 3:** Number of families and EC number types associated with different EFICAz^2 ^predictive components

Type of EFICAz^2 ^component	Three-field EC numbers		Four-field EC numbers	
		
	EFICAz^2 ^version 10	EFICAz^2 ^version 13	EFICAz^2 ^version 10	EFICAz^2 ^version 13
				
PFAM families	2294/**202**^(1)^	2294/**201**	2022/**1987**	2153/**2069**
CHIEFc families	2932/**208**	2947/**209**	3548/**2248**	3607/**2354**
PROSITE patterns	807/**102**	1949/**128**	527/**228**	1368/**437**
All EFICAz^2 ^components	**208**	**209**	**2248**	**2354**

^(1) ^Non-bolded font indicates number of families or patterns while bolded font refers to the number of different EC number types recognized by the indicated category of EFICAz^2 ^predictive component.

### Benchmarking of EFICAz^2 ^version 10

**Figure 6:**
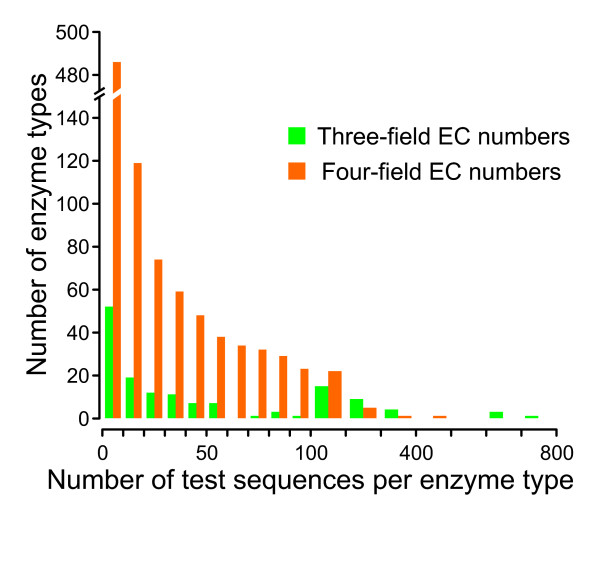
**Distribution of the number of test sequences per enzyme type**. Distribution of 9,397 test enzyme sequences into 145 types of three-field EC numbers (green columns) and 6,996 test enzyme sequences into 614 types of four-field EC numbers (red columns).

To evaluate the effect of the modifications introduced into EFICAz, we performed a benchmark using annotated Swiss-Prot sequences that were not used for training EFICAz^2 ^version 10. First, we generated (as described above) "enzymes" and "non-enzymes" reference sets from all the newly added Swiss-Prot sequences in UniProt Release 12.6 that were not included in the Release 10 of this database. The test sequences used to evaluate three-field EC number prediction performance consist of all the 16,430 members of the "non-enzymes" set plus 9,397 members of the "enzymes" set annotated with at least one of the 208 three-field EC number types recognized by EFICAz^2 ^version 10. Similarly, the test sequences to evaluate four-field EC number prediction performance include the 16,430 non-enzymes plus 6,996 members of the 'enzymes" set annotated with at least one of the 2,248 four-field EC number types recognized by EFICAz^2 ^version 10. Figure 6 shows the distribution of the number of test sequences per enzyme type. Then, we compared the functional annotations of each test sequence in UniProt 12.6 with our functional predictions using EFICAz^2 ^version 10, which is based on the Release 10 of UniProt.

For a given enzyme function ***f ***described by a three-field or four-field EC number, we calculate: **precision_*f *_= TP_*f*_/(TP_*f*_+FP_*f*_)**, and **recall_*f *_= TP_*f*_/(TP_*f *_+ FN_*f*_)**, where (i) **TP_*f *_**(number of true positives) is the number of test sequences for which the function ***f ***is assigned by both EFICAz^2 ^and UniProt 12.6, (ii) **FP_*f *_**(number of false positives) is the number of test sequences for which the function ***f ***is assigned by EFICAz^2 ^but not by UniProt 12.6, and (iii) **FN_*f *_**(number of false negatives) is the number of test sequences for which the function ***f ***is assigned by UniProt 12.6 but not by EFICAz^2^.

In UniProt, as well as and in most protein sequence databases, the distribution of different EC classes is non-uniform, i.e. some enzyme functions are overrepresented while others are underrepresented (see Figure 6). To reduce the bias towards the most represented enzyme functions, we evaluate precision and recall for each individual enzyme function ***f***, and then calculate average values. On the other hand, it is clear that test sequences with higher sequence identity to training enzymes are easier to predict than those exhibiting lower sequence identity. This correlation plus the fact that, in general, the sequence identities of the test sequences to the training enzymes are not uniformly distributed, introduces another potential source of bias. To reduce this second type of bias, we evaluate EFICAz^2^'s performance at different levels of maximal test to training sequence identity (MTTSI). We define MTTSI as the maximal sequence identity between a given test sequence whose predicted function is ***f ***and any training enzyme whose true function is ***f***.

Given a MTTSI interval ***m ***and an enzyme function ***f***, we first select the test sequences whose EFICAz^2 ^predicted function is ***f ***and whose MTTSI falls into the interval ***m***. Then, based on the selected test sequences, we calculate the precision and recall of EFICAz^2 ^for enzyme function ***f ***and MTSSI bin ***m***. For each MTSSI bin, we calculate and report the average precision and recall across all enzyme functions for which these performance indicators are defined (i.e., where (TP_*f *_+ FP_*f*_) > 0 for precision calculation and where (TP_*f *_+ FN_*f*_) > 0 for recall calculation). It has to be mentioned that in previous benchmarks of EFICAz [1,27], we calculated the average precision per MTTSI bin only across the EC number types that were represented in the test sequences. In this work, we decided to average the performance of all possible EC number types, which translates into a decreased average precision (because, by definition, all the additional enzyme functions considered for the average will have zero true positives) but provides a more realistic estimation of our method's performance.

In this work, we evaluated two more versions of EFICAz, besides EFICAz^2^: i) the original implementation of EFICAz where predictions from four component methods are combined without integration by classification tree models, and ii) a version that combines the previous four components and the two new SVM-based components, also lacking the benefit of classification tree predictive models. These versions only differ from EFICAz^2 ^in the number of utilized component methods, or the way the predictions from different components are combined. Thus, the procedures for training of the individual components described above for EFICAz^2 ^also apply to these two other versions of EFICAz.

### Statistical analyses

We performed two-tailed t-tests to determine the significance of the differences in the average recall and precision at specific MTTSI intervals observed between different pairs of predictive methods. Our null hypothesis was that there is no significant change in these performance indicators (critical alpha level = 0.05). To evaluate differences in average recall, we used correlated t-tests because the recall values from each of the two compared methods can be matched according to their specific EC numbers. Conversely, to evaluate differences in average precision, we used t-tests for unpaired data because the prediction precision values associated with each method are not defined for the same set of EC numbers. In this case, assuming that the random variables had different (heteroscedastic t-test) or the same variance (homoscedastic t-test) yielded the same results at the set critical alpha level of 0.05.

### Support vector machine models

We built an SVM model for each particular Pfam and CHIEFc enzyme family, whether the family is associated to a three-field or to a four-field EC number. Each enzyme family consists of a multiple sequence alignment of homo- and hetero-functional members; the goal of each SVM model is to discriminate between them. For classification purposes, homo- and hetero-functional members of an enzyme family are considered as positives and negatives, respectively. To transform the aligned protein sequences into a data matrix suitable for machine learning, a particular amino acid encoding scheme needs to be selected. Several methods for amino acid encoding have been proposed in the literature [38-40]. Here, we adopt an encoding method where each amino acid is represented by five highly interpretable continuous variables derived from multivariate statistic analysis of 494 physicochemical attributes [39]. Thus, for training and evaluation of the SVM models, each aligned position of a member sequence is regarded as a five-dimensional vector, and a multiple sequence alignment with *M *proteins and *N *aligned positions is converted to a data matrix with *M *samples and N*5 input features. Therefore, a different SVM model is associated to each enzyme family, each model having a different number of features, depending on the number of aligned positions. We implemented the SVM models using the libSVM package [41] (kernel function = Radial Basis Function (RBF), γ = 1/k, where k is the number of attributes in the input data, and C = 1).

### Decision tree learning model

Decision trees are predictive models that classify data by mapping features of the data items to inferences about their target values, by means of a hierarchy of questions about such features [29]. Decision trees can be implemented as classification trees when the outcome is discrete, or regression trees when the outcome is continuous [29]. In this work, we have used classification trees to integrate the predictions generated by each of the six EFICAz component methods (C1 to C6) into a final, more precise EC number prediction. The source for training and testing of our classification tree predictive models is the dataset described in "Benchmarking of EFICAz^2 ^version 10", in the Methods section. Our training samples are (**p**, **z**) pairs, where **p **denotes a protein sequence and **z **indicates its EC number. The features considered for the classification are the prediction statuses of the six EFICAz components. We encode the feature information for a given sample (**p**, **z**) in a six dimensional binary vector. Thus, "1" in certain dimension of the vector means that the corresponding EFICAz component predicts that protein sequence **p **exhibits the enzymatic activity associated to EC number **z**, while "0" indicates the opposite. The outcome of the predictive model is a logic variable indicating whether or not **z **is assigned to **p**.

We generated classification trees for two levels of enzyme function description (three- and four-field EC numbers) in two variants each, one for protein sequences with MTTSI < 30% and the other for protein sequences with MTTSI ≥ 30%. The 30% MTTSI threshold was empirically determined and optimized to achieve a biologically useful trade-off between the prediction performance of sequences in or out of the "Twilight Zone" of function prediction, as evaluated in our benchmarks. To create the classification trees, we used the rpart package version 3.1–41 from the statistical analysis tool R [42]. The fitting of the models was done using the default parameters of the rpart function, with the exception of the *weights *argument. We opted for an EC number-dependent case weight equal to the harmonic mean of 1 and 1/N, i.e. 2/(N+1), where N is the number of training sequences that belong to a given EC number. The rationale of this weighting scheme is that it is a halfway balance between two extreme situations: i) implementing a weight = 1/N and thus completely ignoring the natural biases in enzyme abundance that might be partially reflected in databases (all EC number types are treated equally, whether represented by only one or by a large number of sequences), and ii) using a weight ≥ 1 for all cases (no weighting), with the risk of excessively biasing the models towards the EC numbers most abundantly represented in our training set of sequences.

### Enzyme function annotation of the human proteome

The sources for the human protein sequences and their enzyme function annotations were the KEGG Genes and Brite databases (Release 47.0+/06-26, of June 26, 2008), respectively.

## Authors' contributions

AKA and YH participated in the design of EFICAz^2^. AKA conceived of the study, analyzed the results of the performance benchmarks, performed the reannotation of the human proteome, designed the web server and drafted the manuscript. YH implemented the machine learning enhancements of EFICAz^2 ^and helped to draft the manuscript. JS conceived of the study, participated in its design and coordination, and helped to draft the manuscript. All authors read and approved the final manuscript.

## Supplementary Material

Additional file 1 **Figure S1**. Example of Functionally Discriminating Residues (FDRs). Click here for file

Additional file 2 **Novel enzyme function annotations of the human proteome by EFICAz^2^**. Excel spreadsheet listing all the three-field or four-field EC numbers assigned by EFICAz^2 ^version 13 to human proteins that were not annotated as enzymes in the Release 47.0 of the KEGG database. Click here for file
