# Supplementary material for: EFICAz2: enzyme function inference by a combined approach enhanced by machine learning
Source: BMC Bioinformatics. 2009 Apr 13;10:107. doi: 10.1186/1471-2105-10-107 (PMC2670841; doi:10.1186/1471-2105-10-107)
Supplement: Additional file 1 — Figure S1. Example of Functionally Discriminating Residues (FDRs). [file 1471-2105-10-107-S1.pdf]

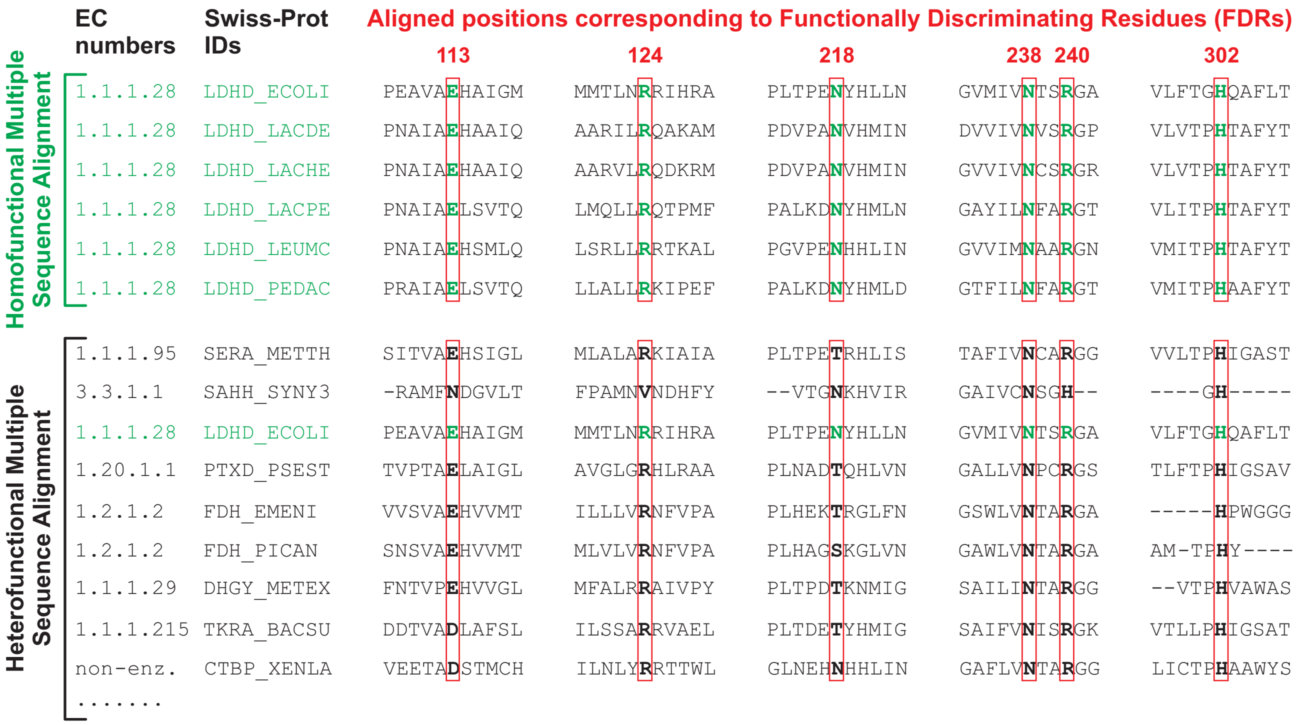

Figure S1. Example of Functionally Discriminating Residues (FDRs). Aligned positions (numbered boxes outlined in red) corresponding to FDRs in the Homofunctional Multiple Sequence Alignment (MSA) and Heterofunctional MSA for an enzyme family whose seed EC number is 1.1.1.28 (D-lactate dehydrogenase). The six FDRs are selected for their ability to discriminate between Homofunctional (i.e., D-lactate dehydrogenases) and Heterofunctional family members (i.e., evolutionary related proteins with any other function). We assign EC number 1.1.1.28 to any sequence recognized as member of the family by a Hidden Markov Model and showing conservation of the six FDRs: E113, R124, N218, N238, R240 (which corresponds to an active site residue in D-lactate dehydrogenases, e.g. active site residue R234 in LDHD_ECOLI) and H302.
